# Antigen Recognition by MR1-Reactive T Cells; MAIT Cells, Metabolites, and Remaining Mysteries

**DOI:** 10.3389/fimmu.2020.01961

**Published:** 2020-08-27

**Authors:** Alexandra J. Corbett, Wael Awad, Huimeng Wang, Zhenjun Chen

**Affiliations:** ^1^Department of Microbiology and Immunology, Peter Doherty Institute for Infection and Immunity, The University of Melbourne, Melbourne, VIC, Australia; ^2^Infection and Immunity Program and Department of Biochemistry and Molecular Biology, Biomedicine Discovery Institute, Monash University, Clayton, VIC, Australia; ^3^ARC Centre of Excellence in Advanced Molecular Imaging, Monash University, Clayton, VIC, Australia; ^4^State Key Laboratory of Respiratory Disease, Guangzhou Institute of Respiratory Disease, The First Affiliated Hospital of Guangzhou Medical University, Guangzhou, China

**Keywords:** mucosal-associated invariant T cell, MR1, antigen, ligand, MR1T, MAIT

## Abstract

Mucosal-associated Invariant T (MAIT) cells recognize vitamin B-based antigens presented by the non-polymorphic MHC class I related-1 molecule (MR1). Both MAIT T cell receptors (TCR) and MR1 are highly conserved among mammals, suggesting an important, and conserved, immune function. For many years, the antigens they recognize were unknown. The discovery that MR1 presents vitamin B-based small molecule ligands resulted in a rapid expansion of research in this area, which has yielded information on the role of MAIT cells in immune protection, autoimmune disease and recently in homeostasis and cancer. More recently, we have begun to appreciate the diverse nature of the small molecule ligands that can bind MR1, with several less potent antigens and small molecule drugs that can bind MR1 being identified. Complementary structural information has revealed the complex nature of interactions defining antigen recognition. Additionally, we now view MAIT cells (defined here as MR1-riboflavin-Ag reactive, TRAV1-2^+^ cells) as one subset of a broader family of MR1-reactive T cells (MR1T cells). Despite these advances, we still lack a complete understanding of how MR1 ligands are generated, presented and recognized *in vivo*. The biological relevance of these MR1 ligands and the function of MR1T cells in infection and disease warrants further investigation with new tools and approaches.

## Introduction

The enormous diversity of possible T cell receptors, combined with the presentation of antigens on polymorphic MHC molecules, enables the detection of a vast number of foreign or altered-self molecules by T cells. Most well-characterized is the specific recognition of peptide antigens by conventional CD4^+^ and CD8^+^ T cells, when presented on MHC Class II and Class I molecules, respectively. Recently, there have been significant advances in understanding antigen recognition by unconventional T cells, in particular natural-killer T (NKT) cells, which recognize lipid-based antigens in the context of CD1 molecules, as well as γδ-T cells, most of which respond to phosphoantigens from infected cells and cancer cells in the context of butyrophilin molecules (1–3). For NKT cells, which share many characteristics with MAIT cells, the first antigen was described in 1997 to be α-galactosylceramide (α-Galcer), derived from a marine sponge (4). Since then, it has become clear that greater diversity exists both in the array of ligands presented by CD1 molecules, and the subsets of NKT cells capable of recognizing these (5, 6).

MAIT cells are a highly conserved unconventional T cell subset, which are abundant in humans and recognize antigens in the context of MR1. MAIT cells express a semi-invariant TCR, comprising TRAV1-2-TRAJ33/12/20 α-chains, paired with a limited array of TCR-β chains (typically TRBV6-1, TRBV6-4, or TRBV20) in humans, and homologous receptors (TRAV1-TRAJ33 paired with TRBV19 or TRBV13) in mice (7–9). The constrained nature of the MAIT TCR repertoire and monomorphic antigen presentation molecule suggested a more limited array of antigens than for conventional T cells. Similar to other unconventional T cell subsets, the MR1T-MR1 axis is being revealed as more complex than initially believed. The first MR1-ligand, the non-agonist 6-formyl pterin (6-FP) was identified in 2012 (10), then some transitory pyrimidine-based MAIT cell antigens were identified in 2014 (11), among which 5-(2-oxopropylideneamino)-6-D-ribitylaminouracil (5-OP-RU) represents the most potent MAIT cell agonist to date. Since then several more MR1 ligands have been described, as well as an increased definition of subsets of MR1-reactive cells, beyond the recognition of riboflavin-based antigens by MAIT cells. Thus, MR1-antigen recognition and MR1-reactive T cell responses in immunity are emerging fields. Like conventional and other unconventional T cell subsets, there is a great promise for developing MR1T-cell-based therapies in several contexts. The definition of the scope of antigens that they recognize, the fine detail of their antigen specificity, and the factors that govern their activation and function will be crucial for achieving this goal.

## MAIT Cell Recognition of a new Class of T Cell Antigen

MAIT cells were initially described over 25 years ago as an abundant CD4^−^CD8^−^ (DN) T cell subset in human blood (8), and later dubbed Mucosal-associated Invariant T (MAIT) cells, due to their conserved TCR usage and enrichment in mucosal tissues, such as the small intestine, in mice and humans (9, 12, 13). It was subsequently shown that MAIT cells reside broadly in tissues like conventional T cells (14) and are restricted to MR1 (12, 15), which has been highly conserved through evolution (16). For several years the antigens recognized through the MR1-MAIT axis were unknown, although early studies suggested they were non-peptide-based molecules (9, 17). The key breakthrough; MR1-ligand identification, was triggered by two key publications from 2010, which demonstrated that MAIT cells could be activated, in an MR1 dependent manner, by a wide range of bacteria and yeasts, but that viruses were non-stimulatory (18, 19). This suggested that the potent activating ligands may come from a conserved biosynthetic pathway and, after careful detective work, this source of antigen was discovered to be microbial biosynthesis of riboflavin (vitamin B2) (10). For a detailed history of the discovery of vitamin B based MAIT cell antigens, readers are referred to previous review articles (20–22) and original research publications (10, 11).

**Figure 1:**
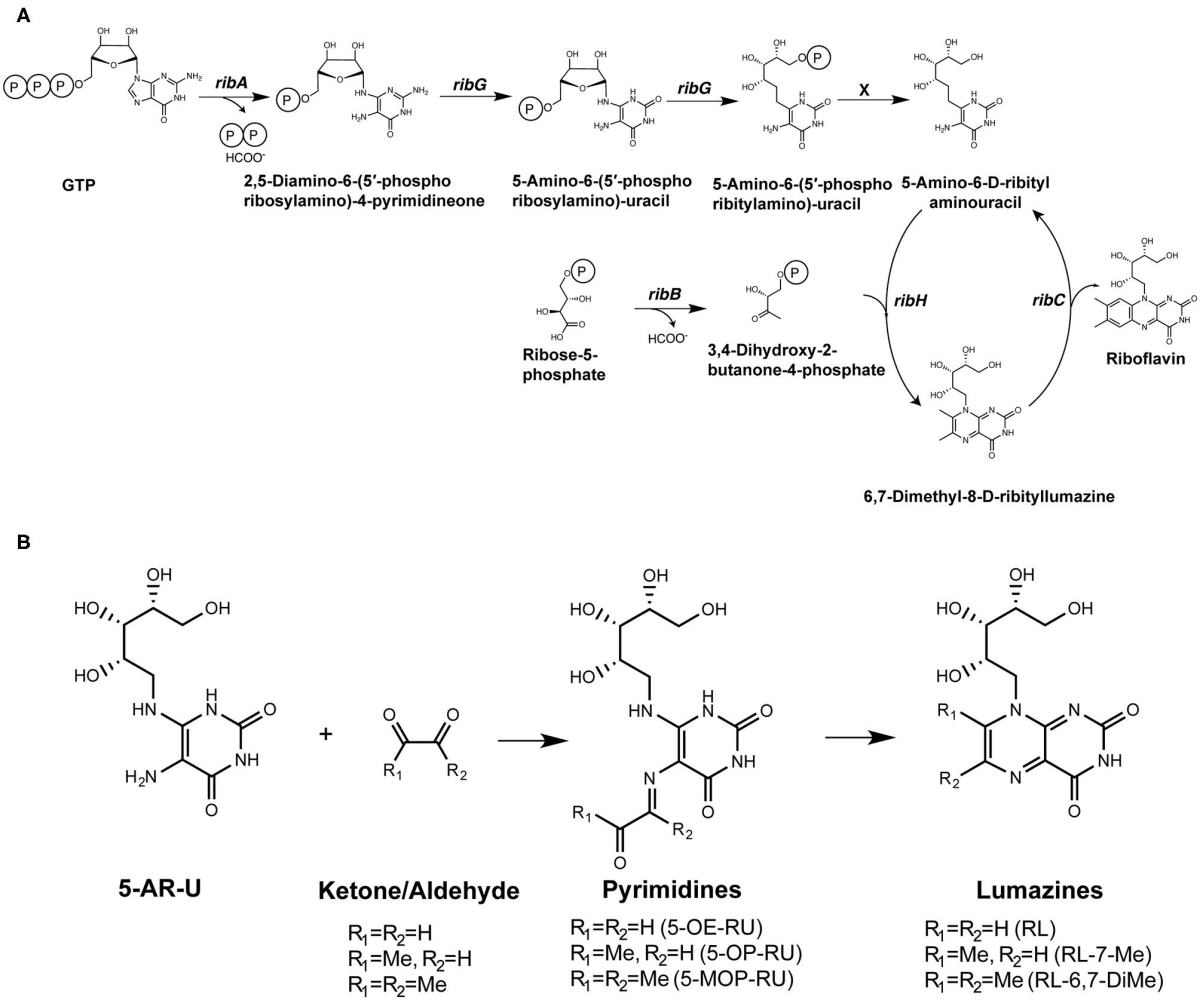
Formation of riboflavin based MAIT antigens. **(A)** Riboflavin biosynthesis pathway. **(B)** The riboflavin biosynthesis intermediate 5-A-RU non-enzymatically reacts with small metabolites to form pyrimidine antigens 5-OP-RU and 5-OE-RU. These can be captured by MR1, or alternatively cyclize to form lumazines, some of which are also weakly antigenic [modified from (11)].

Riboflavin synthesis is a highly conserved biosynthetic pathway, which is essential for many bacteria and yeasts (23, 24). Some organisms cannot synthesize riboflavin but have transporters to take it up from their environment (24), and importantly, several of these microbes (including *Enterococcus faecalis* and *Listeria monocytogenes*) are non-stimulatory for MAIT cells (18, 19). A series of enzymes drive each step in the riboflavin biosynthetic pathway, with *ribA* and *ribG* (alternatively named *ribD* in some microorganisms) being essential for the production of a key intermediate 5-amino-6-D-ribitylaminouracil (5-A-RU). Condensation of 5-A-RU with small carbon metabolites, including glyoxal and methylglyoxal, results in the formation of highly potent pyrimidine MAIT cell antigens 5-(2-oxoethylideneamino)-6-D-ribitylaminouracil (5-OE-RU) and 5-OP-RU, respectively (11) (Figure 1). These pyrimidine antigens are highly unstable, thus are further converted to lumazine derivatives unless trapped by MR1. The derived lumazines, RL-6,7-diMe and RL-6-Me-7-OH, are also capable of activation of human and murine MAIT cells, albeit with reduced potency (10, 25) (Table 1). Studies that identified these novel small molecule antigens utilized an MR1-capture approach, in which recombinant human MR1 was refolded, with human β2 microglobulin, in the presence of culture supernatant from bacteria, such as *Salmonella* Typhimurium, or media controls. Importantly, these metabolite antigens could be detected by liquid chromatography-mass spectrometry (LC-MS) of MR1 refolded in the presence of supernatant from riboflavin-producing bacteria capable of activating MAIT cells, but not from the MAIT cell non-stimulatory bacteria *Enterococcus faecalis*, or *Lactococcus lactis* mutants lacking individual *rib* enzymes (11).

**Table 1:** MR1 ligands identified to date.

**Compound name**	**Chemical structure**	**MAIT activation or inhibition**	**References**
5-OP-RU	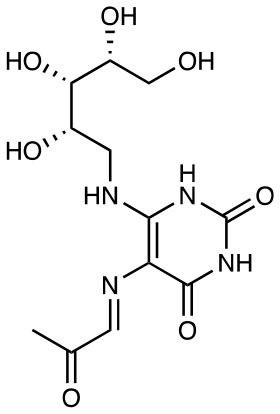	Potent activation of human and mouse MAIT cells EC50 = 1-8 pM	(11)
5-OE-RU	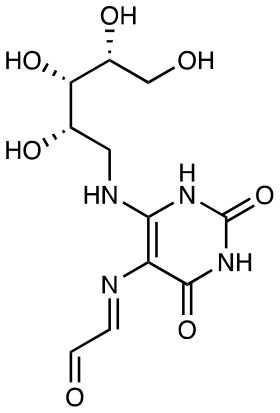	Potent activation EC50 = 510 pM	(11)
RL-6,7-diMe	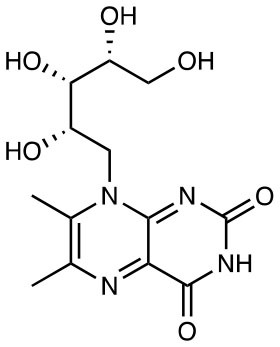	Weak activation	(10)
RL-6-Me-7-OH	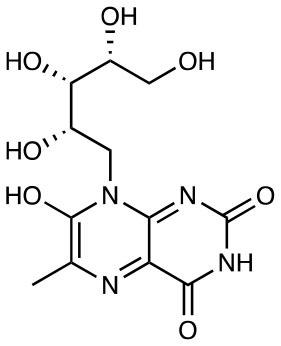	Weak activation EC50 = 25 μM	(10)
6-FP	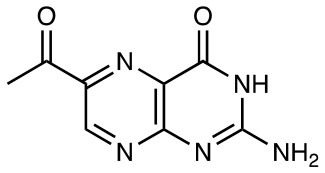	MR1 upregulation of surface expression Competitive inhibition Activation of TRAV1-2^−^ “atypical” MAIT cells	(10) (26)
Ac-6-FP	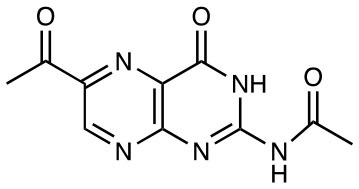	MR1 upregulation of surface expression Competitive inhibition *in vitro* and *in vivo* Activation for TRAV1-2^−^ “atypical” MAIT cells	(25, 27, 28) (26)
2-acetylamino-4-hydroxy-6-formylpteridine	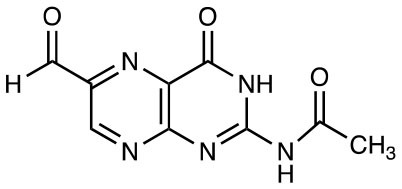	MR1 upregulation Competitive inhibition	(25)
2-acetylamino-4-hydroxy-6-formylpteridine dimethyl acetal	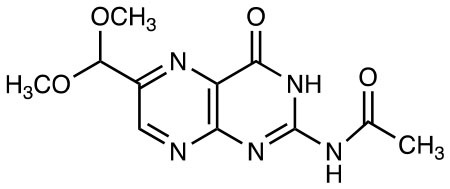	MR1 upregulation of surface expression Competitive inhibition	(25)
Diclofenac (shown) 5-hydroxy diclofenac 4-hydroxy diclofenac Others including: Benzbromarone Chloroxine Floxuridine Galangin Mercaptopurine	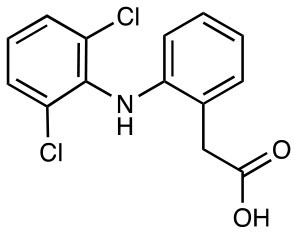	Weakly antigenic with some TCR specificity	(28)
3-formyl salicylic acid (3-FSA) (shown) 5-formyl salicylic acid (5-FSA)	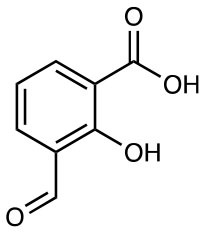	MR1 upregulation of surface expression Competitive inhibition *in vitro* and *in vivo*	(28)
2-Hydroxy-1-naphthaldehyde (2-OH-1-NA) Others including: 1,4 Naphthoquinone 5-Hydroxy-1,4-naphthaldehyde Apigenin Mefenamic acid Menadione	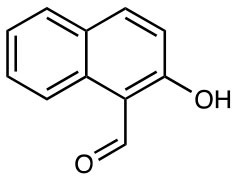	MR1 upregulation of surface expression	(28)
7,8-didemethyl-8-hydroxy-5-deazariboflavin (FO)	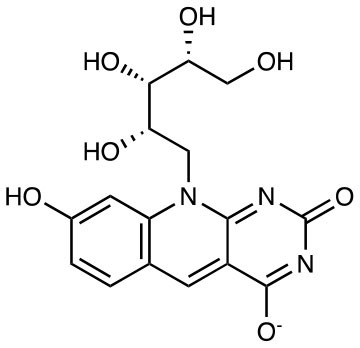	Inhibition of MR1T clone response to *M. smegmatis* supernatant	(29)
6-(1*H*-indol-3-yl)-7-hydroxy-8-ribityllumazine (photolumazine III)	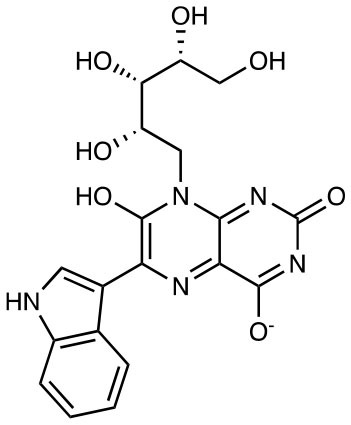	Activation of MR1T clones (blockable by 6-FP)	(29)
6-(2-carboxyethyl)-7-hydroxy-8-ribityllumazine (photolumazine I)	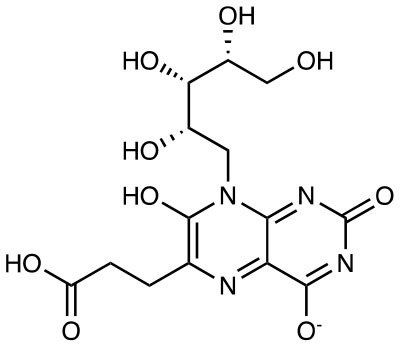	Activation of MR1T clones (blockable by 6-FP)	(29)
3-[(2,6-dioxo-1,2,3,6-tetrahydropyrimidin-4-yl)formamido] propanoic acid (DB28)	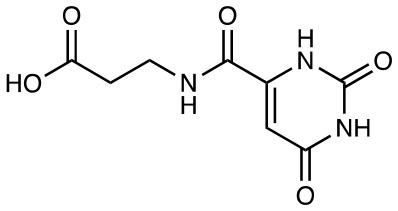	MR1 downregulation of surface expression	(30)

The derivation of the potent antigens, 5-OP-RU and 5-OE-RU, from a metabolic intermediate in a conserved biosynthetic riboflavin pathway, 5-A-RU, appeared to answer the question of why the MR1-MAIT axis has been so highly conserved through mammalian evolution (12, 16, 31) since it suggested that MAIT cells would play an important immune role in protecting against diverse microbial pathogens. The idea that MAIT cells may have evolved to directly detect pathogens (by sensing 5-A-RU-derived molecules) and rapidly respond to metabolically active microorganisms that breach the mucosal barriers was then pursued by many researchers in the field, including our own group. Indeed, the presence of MAIT cells has now been shown to contribute to protective immunity against several pathogens capable of riboflavin synthesis (32–34). More recently, roles in barrier function and tissue repair have also been described (35–40), and this would be consistent with the possible sensing of antigens from microflora, which may indicate a breach of barrier function.

Although the most potent MAIT cell agonist known to date, 5-OP-RU, is often studied or cited in isolation, several MR1 ligands, including both MAIT cell agonists and non-agonists, have now been described (Table 1). Even in the initial discovery studies it was evident that, like for conventional T cells and other unconventional T cells, there was not just one antigen, but a family of related molecules that could bind MR1 and potentially interact with MAIT cells. The question of just how large this MR1-ligand family is, remains. MR1 bound antigens are recognized by MAIT cells through their TCR, which is conserved, but not completely invariant. Thus, it has been hypothesized that different antigens are differentially recognized by MAIT cells expressing different TCRs. Indeed structural studies have demonstrated a role for TCRβ chain in antigen recognition, suggesting that certain subsets of MAIT cells may be enriched in response to different antigens (27). We will address both the diversity of MR1 ligands and the recognition of MR1-antigens by MAIT cells and other MR1-reactive T cells in the following sections.

## Expansion of the MR1 Ligand Family

The first identified MR1-ligand, 6-FP, is a photosynthetic breakdown product of folic acid. This pterin-based ligand is non-stimulatory for most MAIT cells (10) and exhibits a competitive inhibitory effect on MAIT cell activation by 5-OP-RU (25, 27, 28, 41). Acetyl-6-FP (Ac-6-FP) and acetylamino-4-hydroxy-6-formylpteridine dimethyl acetal, synthetic derivatives of 6-FP, are similarly capable of binding to MR1 (as judged by an increase in cell surface expression), but do not activate MAIT cells and, like 6-FP, competitively inhibit activation by 5-OP-RU (25, 27, 28, 41) or *E. coli* (25, 42).

Recently, the family of MR1 ligands has grown significantly, with several groups identifying compounds capable of binding to MR1 and/or stimulating MAIT cells (Table 1). Using *in silico* screens of chemical libraries followed by *in vitro* functional testing, Keller et al. showed that over 20 compounds, with different chemical scaffolds, could bind to MR1 and modulate MAIT cell activity (28). Interestingly, these included some common drugs and drug metabolites such as diclofenac and the salicylates 3-formylsalicylic acid and 5-formylsalicylic acid. This study also demonstrated competitive inhibition of MAIT cell activation *in vivo*, suggesting potentially important physiological effects. Additionally, there was some selectivity in the ability of diclofenac metabolites to activate cell lines expressing MAIT TCRs with the same TCR α-chain but different β-chains, consistent with the role of the β-chain in antigen recognition shown in an earlier study (27).

It is considered likely that additional riboflavin-related antigens may exist. Soudais et al. showed that, as well as methylglyoxal and glyoxal, the small molecule di-hydroxy acetone (DHA) in combination with 5-A-RU, caused activation of mouse MAIT cells, *in vitro*. However, it is unclear whether this was due to the generation of a novel antigen, or conversion of DHA to methylglyoxal (25). In contrast, the same small molecules, glyoxal, methylglyoxal, and DHA, when mixed with 5-nitro-6-D-ribitylaminouracil (5-N-RU) did not significantly activate mouse MAIT cells (25). In 2018, Harriff et al. identified several additional MR1 ligands. These included photolumazines I and III, each capable of activating TRAV1-2^+^ T cell clones and, more weakly, a TRAV1^−^ T cell clone in an MR1-dependent manner and 7,8-didemethyl-8-hydroxy-5-deazariboflavin (FO), which could competitively inhibit activation. They also described the capture by MR1 of the plant flavonoid hesperidin, although this did not appear to either activate or inhibit MAIT cells (29). The reduced potency of these MR1-ligands for MAIT cells (or reporter cells expressing MAIT TCRs) (Table 1), and the selective activation of subsets of MR1T cells, mean it is currently unclear what the physiological role of T cell detection of such molecules plays in immunity.

Most recently, using an *in silico* screen based on the MR1 binding pocket, Salio et al. identified an intriguing effect of a novel MR1 ligand (3-[(2,6-dioxo-1,2,3,6-tetrahydropyrimidin-4-yl)formamido]propanoic acid “DB28,” which appeared to decrease, rather than increase cell surface expression of MR1, as well as competitively inhibiting activation of MAIT cells by agonist ligands (30).

Although many do not appear to be antigenic to MR1-5-OP-RU reactive MAIT cells, it is now widely accepted that the MR1 ligands encompass more than those originally described. Intriguingly, crystal structures (10, 11, 27, 41, 43, 44) show that the known ligands do not fully occupy the MR1 antigen-binding cleft, suggesting that, in addition to small variations, much larger and structurally different scaffolds of MR1-ligands may be possible. Thus, it is likely that the list of described MR1 ligands will continue to grow, and these may also represent antigens for MAIT cells and other MR1T cells.

## Understanding MAIT Cell Recognition of MR1-Presented Riboflavin-Based Antigens

A remarkable characteristic of the potent pyrimidine antigens, 5-OP-RU and 5-OE-RU, and the pterin-based ligands, such as 6-FP, is that these are capable of forming a covalent bond to the MR1 antigen-presenting molecule via a Schiff base formed between the ligand and a Lys residue at position 43 of MR1, which sits in the base of the antigen-binding pocket (10, 11, 45). Whilst several MR1-ligands have now been described, the ability to form this linkage appears critical for stable binding to MR1, as detected by egress of loaded MR1 molecules from the ER and upregulation on the cell surface (44–46). The lower potency of the related lumazine antigens also appears to be due to their inability to form a Schiff base with the Lys 43 residue. Interestingly, the MR1 downregulating ligand, DB28, was unable to form a Schiff based with the Lys 43 residue (30). Within MAIT TCRs, there was the conservation of key residues including a highly conserved Tyr at position 95 of the CDR3 loop of the TCR α-chain. X-ray crystallographic analyses of MAIT TCR-MR1 complexes revealed that this Tyr95α “reaches” down toward the small molecule antigens, potentially explaining the selective TCR usage among MR1-5-OP-RU reactive MAIT cells (11, 27, 41, 47), with the MAIT TCR β chain also playing a role in antigen recognition (27).

Despite these advances, our understanding of the high potency of 5-OP-RU remains incomplete. A panel of 20 analogs of 5-OP-RU was recently developed (44, 48) (**Figure 3**) in order to better understand the intricate factors for MR1 and TCR binding. These “altered metabolite ligands” (AML) are equivalent to altered peptide ligands (APL) which have been instrumental in defining the rules governing classical MHC I and II peptide recognition by conventional T cells. Using the complementary approaches of chemical, functional and high-resolution structural analyses, Ler et al. established a set of molecular rules governing ligand binding to MR1 and interactions with the TCR, driving activation. The impact of various modifications to the antigen correlated with the extent to which they disrupted the formation of an “interaction triad,” a network of hydrogen bonds between the conserved Tyr95α in the CDRα loop of MAIT TCRs, the ribityl moiety of the antigen and the Tyr152 residue of MR1. MAIT cell activation potency was found to be orchestrated by dynamic compensatory interactions within this “interaction triad”. Among all tested ligands, 5-OP-RU, the most potent MAIT agonist identified to date, was found the most capable of establishing a strong and stable interaction triad. Even small modifications to the ribityl chain resulted in profoundly reduced potency in cellular assays.

**Figure 2:**
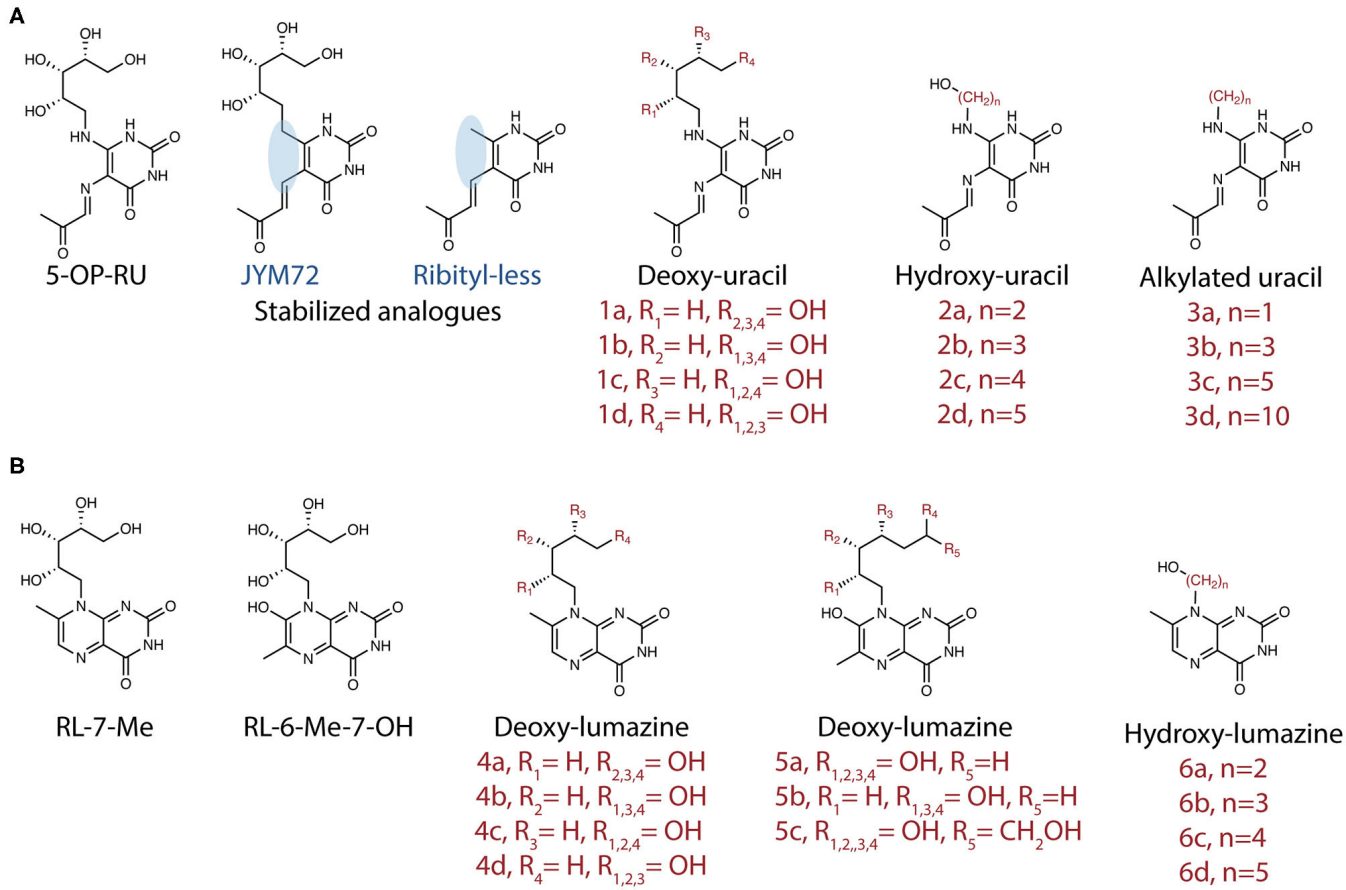
Synthetic analogs of **(A)** pyrimidine antigen 5-OP-RU or **(B)** lumazines RL-7-Me and RL-6-Me-7-OH have been created to understand how different modifications impact MR1 binding and MAIT cell activation or inhibition.

Similar conclusions were reached by a second group, who produced a partially overlapping set of 5-OP-RU analogs (Figure 2A) by different methodologies (49). In both studies, removing the terminal hydroxyl group of the ribityl chain significantly reduced the ability of the ligand to engage the TCR and activate MAIT cells. With this interaction removed, the ligands could act as competitive inhibitors (49). In another study, sugar analogs of the weaker lumazine ligands RL-6-Me-7-OH were also shown to bind MR1 and tetramers loaded with these analogs could stain a cell line expressing a MAIT TCR (50) (Figure 2B). However, their potency in stimulating bone-fide MAIT cells has not yet been examined.

## More than MAITS; The Increasing Diversity of MR1-Reactive T Cells

**Figure 3:**
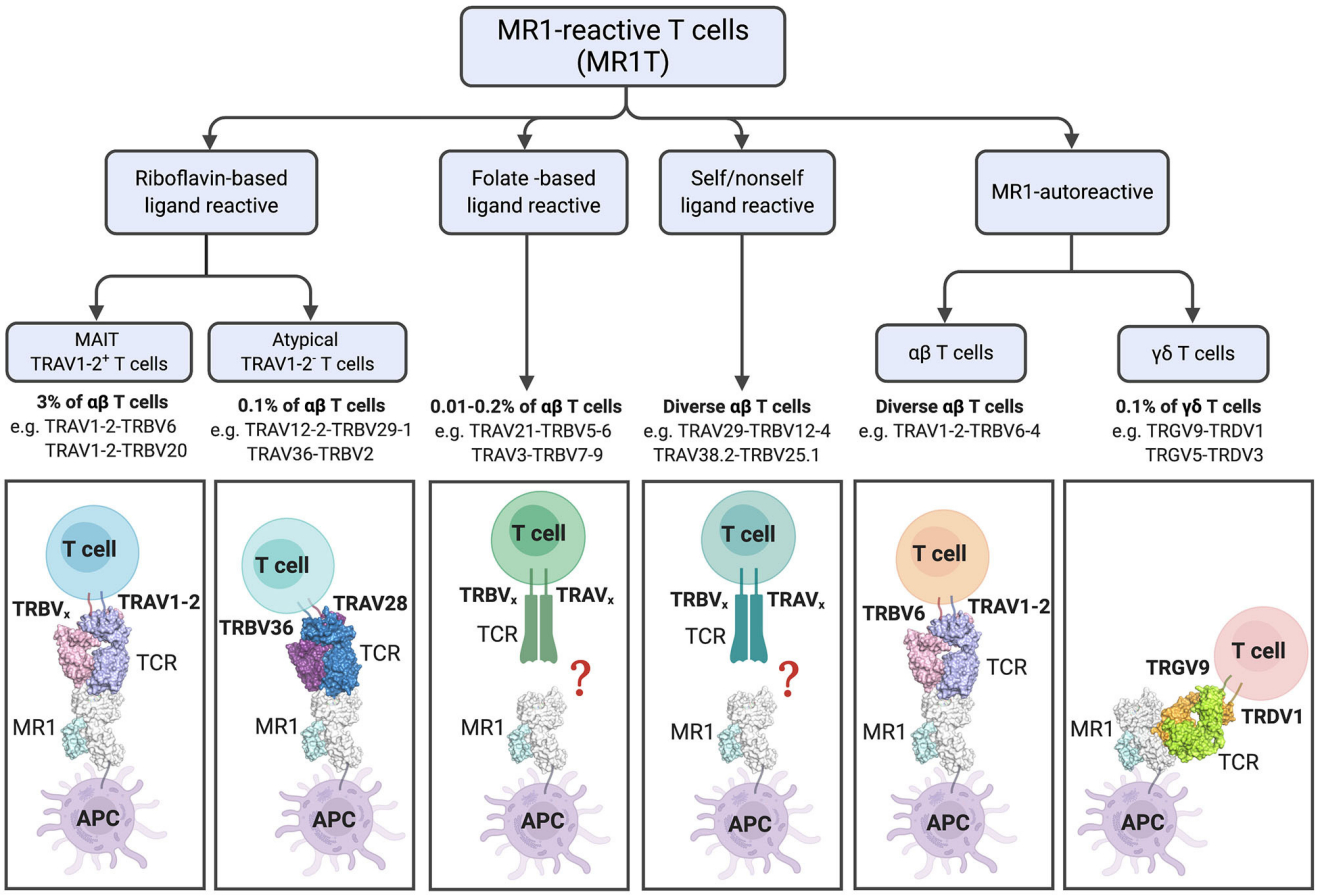
Diversity and characteristics of MR1-reactive T cells. % refer to those reported in human PBMCs. For subsets with diverse TCRs examples are listed. The lower panel shows cartoon representations of the crystal structures of ternary TCR-MR1-Ag complexes: The typical MAIT A-F7 TRAV1-2-TRBV6-1 TCR-MR1-5-OP-RU (PDB; 6PUC); the atypical TRAV36-TRBV28 TCR-MR1-5-OP-RU (PDB; 5D7L); MR1-autorective M33.64 (TRAV1-2/TRBV6-4) TCR-MR1-5-OP-RU (PDB; 5D5M); and G7 γδ TCR with MR1-5-OP-RU complex (PDB; 6MWR). The MR1 and β2-microglobulin molecules are colored white and pale-cyan, respectively. TRAV1-2 TCRα, light-blue; TRAV6 TCRβ, light-pink; TRAV36 TCRα, violet-purple; TRBV28 TCRβ, sky-blue; TRGV9 TCRγ; lemon, TRDV1 TCRδ, orange. Structural illustration was created using PyMOL Molecular Graphics System, Version 1.8.6, Schrodinger and BioRender.com.

MAIT cells were initially described as a large population of cells with restricted TCR α- and β-chain usage (8, 9). This, together with the high conservation of MR1 (51) suggested a capacity for recognition of a single or limited set of antigens. However, as described above, the array of MR1-ligands that may serve as T cell antigens is now acknowledged to be larger than first believed (Table 1). Development of MR1-tetramers in 2013 (K43A mutant MR1) and 2014 (WT mouse and human MR1) enabled sorting and TCR sequencing of MR1-5-OP-RU reactive cells. This revealed greater heterogeneity of TCR usage than previously described (7); a finding later supported by other studies (52–54). We define MAIT cells here as TRAV1-2^+^ MR1-riboflavin-Ag-reactive T cells (Figure 3). These cells can be detected using MR1-5-OP-RU tetramers, are restricted to MR1, acquire the hallmark promyelocytic leukemia zinc finger (PLZF) molecule during thymic development (55), and display an effector-memory phenotype (CD44^hi^ and CD62L^lo^).

In addition to the riboflavin-reactive TRAV1-2^+^ MAIT cells, populations of TRAV1-2^−^ MR1-reactive T (MR1T) cells have been described by several groups. In 2016, Gherardin et al. described “atypical MAIT cells,” which were variably riboflavin- or folate- reactive or MR1 autoreactive cells (26), with a role for the CDR3β loops of the TCRs in determining reactivity. These cells were relatively rare, comprising <0.1% of αβ-T cells in human blood. Additionally, more diverse TRAV1-2^−^ cells were described, which were phenotypically distinct from TRAV1-2^+^ MAIT cells. X-ray crystallographic analyses of one TRAV1-2^−^ (TRAV36-TRBV28) TCR in complex with-MR1-5-OP-RU revealed a different mode of recognition, whereby the TCR docked more centrally on MR1 compared to the TRAV1-2^+^ TCRs [reviewed in detail (56)] (Figure 3). Broadly consistent with these studies, another group reported the existence of MR1-5-OP-RU reactive TRAV1-2^−^ cells, with at least one clone instead expressing TRAV12-2, which lacks the Tyr95 residue previously found to be conserved in TRAV1-2^+^ MAIT cells (57). Furthermore, this clone displayed distinct antigen specificity, detecting infection with *Streptococcus pyogenes* (group A strep), which does not have the capacity to produce riboflavin, suggested recognition of microbial non-ribityl-based antigens (57). Similarly, a proportion of cells staining with MR1-tetramers generated with *E. coli*-derived ligands were TRAV1-2^−^, again indicating that not all MR1T cells are TRAV1-2^+^ MAIT cells (29). Lepore et al. and Koay et al. have also identified MR1T cells in healthy individuals, which were capable of reacting to MR1-expressing cells in the absence of microbial ligands. These MR1T cells were found to have a diverse TCR repertoire and functional capacity (58, 59).

In addition to αβ-T cells, γδ-T cell populations displaying reactivity to MR1 have recently been described. Interestingly, a distinct population of these γδ-T cells recognized MR1 via a novel binding mode, whereby the TCR binds the underside of the MR1 molecule (60) (Figure 3). Thus, in addition to its role in presenting microbial antigens for specific recognition by MAIT cells, it appears that MR1 may act as a pattern recognition receptor, with its cell surface upregulation potentially triggering a more innate-like immune response. It is unclear whether MR1 molecules are present at the cell surface in an empty form, but it is considered likely that they contain endogenous (self) ligands. Indeed, recent studies suggest MR1 can present endogenous or tumor antigens to MR1T cells other than MAIT cells (58, 61). However, these antigens have not yet been identified.

Thus, our definition of MR1-reactive T cells has expanded to encompass not just MR1-5-OP-RU reactive TRAV1-2^+^ MAIT cells, but folate-reactive and autoreactive atypical MAIT cells, as well as γδ-T cells (Figure 3). Thus, the term MR1T cells is now used to denote all MR1-reactive cells, of which MAIT cells represent the majority. In contrast, other subsets of MR1T identified to date are PLZF negative. While the co-evolution of MR1 with the TRAV1 invariant TCR alpha chain (16, 62) suggests that presentation of microbial signature Ag to MAIT cells is the primary function of MR1, questions remain regarding the thymic development, functional capability and physiological relevance of the other MR1T cell populations. Although some subsets, such as the folate-reactive “atypical MAIT cells” represent only a very small percentage, as pointed out by Gherardin et al., these are present in similar numbers in humans to type I NKT cells, and may be expanded in disease settings (26). The selective recognition of antigens by various subsets of MR1T cells expressing different TCRs suggests that these unconventional T cells may generate display specific memory cell pools similar to conventional T cells. Indeed, a recent study by Loh et al. demonstrated that older adults have large clonal expansions of MAIT cells, similar to those seen in conventional virus-specific CD8^+^ T cells (63). Clonal expansion of MAIT cells has also been reported (64) and these may suggest differential recognition of diverse MR1-ligands, or expansion of higher affinity clones in some individuals, perhaps due to a history of infection. A recent study of a small cohort of patients with multiple sclerosis reported oligoclonality, with limited TCRβ repertoires, which remained stable over 3 years (65). Stable oligoclonality was confirmed in healthy individuals by Howson et al., who also found in six individuals the MAIT TCRβ repertoire maintained oligoclonality following *S*. Paratyphi infection, but with expansion and contraction of particular clonotypes, resulting in an altered composition. Cell lines expressing TCRs from expanded clones were more responsive to MAIT cell riboflavin-based antigens (66) suggesting preferential expansion of activated MAIT cells. The TCR repertoire of expanded cells has not yet been assessed in experimental settings such as in mouse models using defined antigens. Since the selective recognition of MR1 ligands by subsets of MR1T cells, potentially with functional differences, present opportunities for selective targeting, more research is needed into this area.

## MR1-Antigen Recognition in Physiological Settings

Although several advances have been made in defining MR1-ligands and their recognition by MR1T cells, many questions remain about the physiological relevance of each antigen. As outlined recently by Ler et al. (44), the ability of ligands to generate a MAIT cell response is the result of the combination of several factors, including chemical properties of the ligands, the ability to bind strongly to MR1 resulting in egress to the cell surface, and the avidity of T cell recognition. The expression and regulation of MR1-ligand presentation remain incompletely understood, and this will impact the activation of MR1-reactive T cells *in vivo*. It is clear from studies comparing different natural ligands or altered metabolite ligands (AML), that the recognition and subsequent response depends on many factors, including chemical stability, binding to MR1 (significantly dependent on the ability to form a Schiff base covalent bond) and TCR recognition (27, 41, 44, 46). To date, 5-OP-RU is the most potent MAIT antigen in any assay, activating MAIT cells even at pmol concentrations. It has also been shown to be relevant to MAIT cell thymic development and activation of MAIT cells *in vivo*. On the other hand, a key question remaining in the field is whether any of the less potent antigens, antigens that activate smaller subsets of MR1T cells, or MR1 ligands that inhibit MAIT cell activation, are physiologically relevant. Here, we will focus not on the MRIT cell response in these settings, but clues to the *in vivo* production and MR1-presentation of antigens in different contexts.

### Physiological Expression and Impact of MAIT Antigens

Riboflavin-based antigens that stimulate MAIT cells include the potent pyrimidine-based compounds 5-OP-RU and 5-OE-RU, have now been identified from several bacterial species, including *Salmonella* Typhimurium, *Escherichia coli, Lactococcus lactis*, and were not detected from riboflavin deficient bacteria (*E. faecalis*) (10, 11). Indeed, bacteria with deletions in the *rib* genes necessary for this biosynthetic pathway are incapable of a full *in vivo* response, as MAIT cells accumulated in the lungs after intranasal infection with *Salmonella* Typhimurium but not a *ribDH*-deficient mutant (67). Many other microbes possessing the riboflavin pathway have now been shown to activate MAIT cells [summarized in a previous review (20)] and the requirement for the presence of the riboflavin pathway in this activation was confirmed for *E. coli* (25) and *S. pneumoniae* (68), as well as in a study screening 47 microbiota-associated bacterial species. In the latter study, differences in activation capacity were observed between different phyla, and levels of riboflavin production correlated with MAIT cell activity (69). The presence of shared antigens, 5-OP-RU and 5-OE-RU by these microbes is likely, although so far these have only been detected for a handful of bacterial species. Additionally, the physiological relevance of the less potent, but related, lumazines particularly during infection, has not been fully addressed.

MAIT cells have been analyzed in several mouse models of bacterial infection, demonstrating their role in protecting against pathogenic organisms such as *Francisella tularensis* (33, 70), *Klebsiella pneumoniae* (32), *Mycobacterium bovis* BCG (54), and *Legionella longbeachae* (34). Riboflavin-based antigens are likely produced not only by pathogenic microorganisms during infection, but also by commensal microorganisms. Indeed, the microbiota is important for the development of MAIT cells, as they are almost completely absent in the periphery in germ-free mice (12, 55). The re-introduction of bacteria, even with single strains, was shown to restore MAIT cells (19). Recently it was demonstrated that the ability of microflora to support thymic development of MAIT cells was dependent on riboflavin biosynthesis, and that providing exogenous 5-OP-RU was sufficient to reconstitute MAIT cell development (71). Recent studies also demonstrate the importance of gut microbiota and the presence of *rib* genes, on MAIT cell reconstitution after allogenic haematopoietic cell transplantation in humans (72, 73). Dysbiosis in viral infections or changes to the gut microbiota have been shown to be associated with impaired MAIT cell responses (74) and it is hypothesized that dysbiosis in metabolic conditions also affects MAIT cells, altering their capacity to promote barrier integrity, and instead triggering a pathogenic, inflammatory MAIT cell response (75). Understanding microenvironmental factors controlling the production of antigen by both pathogens and commensal microorganisms will be important for a complete understanding of MAIT cell responses.

Despite an abundance of information on the regulation of riboflavin production in several bacteria and fungi (23, 24), and the identification of 5-A-RU as the key chemical building block of these molecules, the *in vivo* production of riboflavin-based antigens, including 5-OP-RU and 5-OE-RU (11), remains poorly understood. For example, recently Kurioka et al. showed that the *S. pneumoniae* riboflavin operon genes, which are highly conserved between *Streptoccci*, were upregulated with heat stress, followed by later downregulation (68). It is thus probable that MR1-antigen production, and the resultant MAIT cell activation, may be enhanced under different conditions that may be encountered during the course of infection. Schmaler et al. showed that the growth of bacteria in conditions designed to resemble the human colon (e.g., low oxygen) or on different sugar sources affected bacterial metabolism and the subsequent ability of bacterial samples to activate MAIT cells (76). Intriguingly, the ability of *E. coli* to activate CD8^+^ human peripheral blood MAIT cells, *in vitro*, was increased by treatment with the pesticides chlorpyrifos and glyphosate, which also were found to cause changes in the riboflavin and folate biosynthesis pathways (42). Moreover, folate producing bacteria that didn't stimulate MAIT cells (*Lactobacillus reuteri* and *Bifidobacterium adolescentis*) were shown to inhibit activation of human MAIT cells by *E. coli*, as measured by the production of IFNγ and TNF (42), suggesting the balance of activating and inhibitory MR1-ligands may be important for the generation of MAIT cell responses, *in vivo*.

In humans, MAIT cell responses during infection have been suggested by studies of MAIT cells in patient cohorts, including in the blood and an increasing selection of tissues, paired with subsequent *in vitro* analysis. These studies included patients with tuberculosis, *Helicobacter* infection, *cholera*, and undefined infections such as sepsis and community-acquired pneumonia (77–80). MAIT cells were also activated in human volunteers infected with live *Salmonella enterica* Paratyphi A vaccine (66). The role of human MAIT cells in infection and diseases has been reviewed elsewhere (14, 81, 82) so will not be covered in detail here.

In addition to the setting of infection with pathogens producing riboflavin-based antigens, MAIT cells have been studied in the context of viral infections, autoimmune diseases including diabetes, multiple sclerosis systemic lupus erythematosus and arthritis, and have most recently gained attention as potentially important in cancer. There have been several recent reviews covering the findings in these areas as well as animal models developed, which allow the study of MAIT cells in these settings (75, 83–87). The function of MAIT cells in these settings is complex and differs from the direct anti-bacterial role seen in infection models. For example, in the non-obese mouse model of diabetes, deletion of MR1, and thus MAIT cells, resulted in a loss of gut integrity, indicating a protective function, but MAIT cell alterations, including increase in cytotoxic effectors were also observed in mice and human type-1 diabetic patients at the onset of disease suggesting a pathogenic role (35). A role for MAIT cells in tissue repair or inflammatory conditions such as inflammatory bowel disease would be consistent with their enrichment at barrier sites where they may encounter metabolically active pathogenic or commensal microorganisms. However, the recognition of riboflavin-based or other MR1-ligands in these settings has not yet been confirmed.

### MR1 Expression and Regulation

MR1 is highly conserved and shows low genetic polymorphism (16). The MR1 protein is expressed, along with β2m, at low to undetectable levels on a range of cell types (88). MR1 is ubiquitously expressed at the transcript level in mouse and human cells (31, 89), although recent studies point to different levels of expression in different tissues (90, 91). Like MHC Class I molecules, MR1 forms stable complexes on the cell surface only when stably folded in the presence of a ligand (88, 92) and upon ligand binding MR1 is released from the ER and traffics to the cell surface (45), a characteristic exploited for detecting the presence of ligands in cellular assays of MR1 upregulation (10, 11, 45). However, MR1 remains difficult to analyse due to its low expression, particularly in primary murine cells, despite evidence for an its function both in MAIT cell development (12) and impressive MAIT cell accumulation *in vivo* (67).

Several cell types have been used as MR1 antigen-presenting cells (APCs) in *in vitro* assays. *In vivo*, one recent study showed that both bone marrow-derived professional APC and non-bone marrow-derived cells (epithelial or stromal cells) can present antigen to activate a MAIT cell response, with the relative importance determined by the biology of the infecting pathogen (93). Thus, it remains to be fully understood which cells are responsible for MAIT cell activation in physiological settings such as during bacterial infection.

Downregulation of MR1 by HSV-1 and CMV suggests that TCR-dependent MAIT cell activation may also be impaired in the context of viral infection (94). Given that MAIT cell numbers and function are altered in human cohorts including bacterial infection or sepsis, viral infection and autoimmune disease, it remains to be shown whether there is a direct link to MR1 expression. Seshadri et al. identified a single nucleotide polymorphism in an intronic region of MR1, which was associated with susceptibility to tuberculosis in a Vietnamese adult cohort. This SNP was also associated with MR1 expression in cell lines, and thus may act to modulate the MR1-dependent response of MAIT cells (95). MR1 also exists in multiple isoforms (31) but the biological relevance of these is unclear.

## The Context of Antigen Recognition Determines MAIT Cell Responses

MAIT cells have been described as “innate-like” rapid responders, with a memory-like phenotype. However, like conventional T cells, MR1-antigen alone is insufficient to drive an optimal MAIT cell response, with co-stimuli including cytokines and/or surface co-receptors required (67) and able to enhance MAIT responses to antigen stimulation (96–99), although *in vitro* findings have not always translated to *in vivo* studies (70). Additionally, polarizing effects on MAIT cell function have been demonstrated. In particular, priming with IL-7 or treatment with IL-23 significantly increases IL-17 secretion, which otherwise is minimally induced in MAIT cells (93, 100, 101). IL-7 has been linked to effects on MAIT cell function and polarization in disease settings such as multiple sclerosis (102) and primary biliary cholangitis (103). Recently, it was shown that ICOS and IL-23 are important for MAIT cell *in vivo* responses in mouse infection models with *Salmonella* and *Legionella* (93). In genetically deficient mice lacking these co-stimulators, expansion of MAIT cells was impaired and the profiles of responding pulmonary MAIT cells shifted from RORγt^+^ to T-bet^+^ phenotypes (93).

Different cytokine signals synergize to control MAIT responses (104), and thus, the MAIT cell functional capacity may differ in tissue settings compared to blood MAIT cells (105). In humans, most peripheral blood MAIT cells display type-1 responses upon stimulation, whereas in tissues a IL-17-secreting population is more readily detected (80, 105), suggesting there may be functional differences. Following infection with *S*. Paratyphi, the MAIT cell response was partially IL-12-dependent, suggesting the quality and quantity of MAIT cell immunity is driven by the combination of pathogen signals and host cytokines (66). The augmenting effects of cytokines may act via increasing expression of the MAIT TCR (101) or upregulating cellular components related to TCR signaling (100).

In the absence of TCR-dependent antigen recognition, MAIT cells can be activated by cytokines, including IL-12 and IL-18 (54, 106–108), and by super-antigens (109). In one study, the MAIT cell response in group A streptococcus (GAS) infection appeared to contain both TCRβ-dependent (super-antigen) and independent activation (110). Cytokines likely drive the MAIT cell activation observed in viral infections and contribute to their response during bacterial infection (111). Importantly, there are qualitative and quantitative differences in the MAIT cell responses triggered by TCR-dependent and TCR-independent mechanisms (38). However, the implications of these differences in various immune settings are yet to be determined.

In settings of chronic inflammation, infection or cancer, MAIT cells display altered phenotypes (112), can produce Th2 cytokines (113), and can contribute to pathology (114). MAIT cell function may be impaired in settings such as chronic infection (115–117) and cancer (118). Various studies have implicated immune checkpoints (116, 119, 120), cytokines such as IL-10 (121) and suppression of MAIT cell function by hydrogen peroxide released from neutrophils (122). Thus, in addition to recognition of MR1-bound antigen, the context of other signals in different setting is important for MAIT cell activation and function.

## Tools for the Assessment of MR1 Ligand Production and Recognition

The discovery of a new class of antigens, while providing exciting opportunities, also presents challenges when seeking to understand their biological significance. The study of metabolite-based ligands presents new and different challenges to the better-known peptide and lipid-based antigens. The MR1-binding and MAIT cell activation by metabolites of drugs such as diclofenac (28), as well as the recently described breakdown of 5-A-RU in air (123), highlights the importance of confirming the precise identity of MR1 captured ligands by a combination of assays such as high accuracy mass spectroscopy, X-ray crystallography, NMR spectroscopy, functional assays and ultimately *in vivo* studies. Identification of 5-OP-RU and 5-OE-RU was originally achieved by mass spectrometric analysis of recombinantly expressed MR1 protein refolded in the presence of bacterial supernatant to “capture” the small molecule ligands, in combination with X-ray crystallography and functional validation (10, 11, 20). These techniques do not easily lend themselves to the detection of MR1-bound ligands from complex biological samples, and to date, the field has not developed a simple assay to confirm antigen identity. Thus, many studies did not confirm the identity of the antigens involved. Evidence of riboflavin pathway involvement provided by detecting or deleting *rib* genes has been used as an indirect confirmation. However, it is possible that 5-A-RU recombines with other small molecules to form alternate antigens. For example, Harriff et al. suggest that novel antigens photolumazines I and III may form from 5-A-RU and α-ketoglutarate, the availability of which may be altered in nitrosative stress during intracellular infection (29).

The MR1 autoreactivity shown by some MR1T cells also reveals the importance of validating reactivity with specific controls (26, 60). In this section we present a summary of the basic tools and approaches used to develop our current understanding of the immune response to metabolite antigens.

### Detection of MAIT Cells Using Antibodies or Tetramers

The precise identification of MAIT cells was hampered for several years, particularly in mice and other non-human mammals, by the lack of specific reagents for their detection. Human MAIT cells can be identified by co-staining with a panel of antibodies. Typically, CD3, CD161, and TRAV1-2 (Vα7.2) are used (124, 125), although some groups have also used the high expression levels on MAIT cells of surrogate markers such as CD26 and IL-18R (13, 57, 126, 127). The CD4^+^, CD8^+^, and double-negative (DN) MAIT cell subsets have been variably included or excluded. However, it is now clear that MAIT cells contain all of these subsets (128). Complicating these approaches, it is apparent that the expression of some markers may change in different physiological settings. For example, CD161 expression decreased in rheumatoid arthritis (129) and HIV infection (121). In mice, there are currently no available antibodies recognizing the TCR Vα19 utilized by mouse MAIT cells. This, combined with the low MAIT cell numbers in naïve laboratory mice, prevented the systematic assessment of MAIT cells in wild-type laboratory mice prior to the generation of MR1-tetramer reagents (7, 11, 54, 67, 130).

The first generation of MR1-tetramers (7) utilized a modified human MR1 containing a Lys to Ala mutation at position 43 at the base of the MR1 A' binding pocket. This mutation enabled the refolding of “empty” MR1-β_2_m molecules in the absence of an added ligand, which would otherwise be needed to stabilize the MR1-β_2_m complex. These K43A MR1-β_2_m monomers could then be loaded with an antigen of choice and tetramerised with fluorochrome-coupled streptavidin, enabling the detection of MAIT cells in human blood and intestinal cell preparations (7). The elucidation of the formation of 5-OP-RU and 5-OE-RU from 5-A-RU and methylglyoxal or glyoxal, respectively, enabled the subsequent generation of mouse and human [and later macaque (131)] wild-type MR1 tetramers loaded with each of these molecules. These were formed by refolding the MR1 and β_2_m proteins in the presence of the precursor molecules, followed by biotinylation and tetramerization by standard methods (11). The resultant new generation tetramers were far more stable and easier to use in standard flow cytometry protocols. These reagents have now enabled the detection and study of MAIT cells in many contexts, similarly to the use over many years of MHC Class I (132) and CD1d tetramers (133). MR1 tetramers loaded with 6-FP and Ac-6-FP were similarly generated. These are typically used as control reagents but have been shown to detect a small number of reactive or MR1-autoreactive cells (26), the biological relevance of which is currently unclear.

Despite the clear overlap between MAIT cells detected using MR1-5-OP-RU tetramers and using antibodies to TRAV1-2 [~95% in human blood (11)], these reagents will not detect all MR1T cells, since some of these may have different antigen specificities. This may be particularly true in tissues, or in different mammalian species, where analysis has been less extensive. Thus, similar to the methods for detecting MR1-ligands discussed above, the generation of MR1-tetramers loaded with different sources of metabolite molecules (29) will likely be useful in understanding MR1T cells other than 5-OP-RU-reactive MAIT cells. For small modifications to 5-OP-RU, such as with the altered metabolite ligands discussed above (44), the identified populations almost completely overlap, suggesting that either MR1T cells recognizing ligands other than 5-OP-RU are rare, or were excluded from this analysis by prior gating. Thus, removing the assumption of shared markers, and the use of tetramers loaded with more complex sources of potential ligands is expected to broaden the scope of detection of MR1T cells.

### *In vitro* Cellular Assays

In order to identify MR1-bound ligands that are recognized by MAIT or other MR1T cells, several groups have developed cellular assays for screening. These utilize either T cell clones or T cell lines (such as Jurkat, SKW3, or mouse 6C2) engineered to express a MAIT TCR, which accordingly acts as a reporter cell line. These are co-cultured with cell lines expressing or overexpressing MR1, which act as antigen-presenting cells. Again, various cell lines have been utilized by different groups, in some cases with MR1 deficient cell lines acting as controls (25, 34, 49, 57, 76, 134). Alternatively, gated MAIT cells within PBMCs are analyzed after stimulation, either alone, or with cell lines or dendritic cells as APCs (11, 28, 46, 124, 135–137). Although a number of studies have examined the *in vitro* activation responses of MAIT cells sourced from human tissues (77, 100, 138, 139) to our knowledge these have not been used for MR1 ligand identification purposes. The activation of cells in these assays has been assessed by upregulation of markers [e.g., CD69, CD25, CD137(41-BB)], down-regulation of CD3 and cytokine production. These readouts give an indication of the presence of activating MR1-ligands, but do not reveal their identity. The presence of MR1-ligands is also indicated by the upregulation of cell surface MR1, as detected by conformational monoclonal antibodies 26.5 or 8F2.F9 (15, 140). However, this measure has some caveats, including lower sensitivity than MAIT cell activation, and the increase in surface expression can be missed depending on the timing of the assay due to the instability of the compounds or MR1-ligand complexes.

Interestingly, 5-A-RU, which is not believed to be a MAIT cell antigen in its own right, can activate MAIT reporter cells in culture, indicating the formation of antigens, such as 5-OP-RU, by reacting with aldehydes or ketones from the medium (such as methylglyoxal) or within cells (11). This highlights the danger of inferring antigen identity when using whole-cell assays to test for MAIT cell activation. To limit the complexity relative to the cell systems, plate-bound MR1 has also been utilized in a similar activation assay (141). The direct elution of MR1-bound ligands from immunoprecipitated cell surface MR1 for identification by mass spectrometry is technically challenging, but has been shown using C1R cells overexpressing MR1 (C1R.MR1 cells) for Ac-6-FP, 3-F-SA and diclofenac metabolites (28).

### Chemical and Biochemical Investigations of MR1 Antigens and Analogs

The small molecules 5-OP-RU and 5-OE-RU are formed as chemical intermediates in the reaction of 5-A-RU with methylglyoxal and glyoxal, respectively. These open-ring compounds rapidly cyclise to form relatively weak lumazine antigen RL-7-Me, and RL (Figure 1). Their original identification as MR1 ligands was achieved by refolding recombinantly expressed MR1 in the presence of synthetic compounds or complex sources of antigen, such as bacterial culture supernatant (10, 11, 28), which was demonstrated to be sufficient to stimulate a MAIT cell response (47). This method enabled the MR1 molecules to “fish out” ligands that were capable of binding in a relatively unbiased approach, for subsequent analysis by LC-MS, which was complemented by high-resolution crystal structures. Bacteria with *rib* gene deletions (*S*. Typhimurium, *L. lactis*) were generated to confirm the involvement of the riboflavin synthesis pathway (10, 11). The relatively short half-life of 5-OP-RU in aqueous solution [t_1/2_ half-life time (t_1/2_) ~1.5 h, 37°C] (11, 46) suggests that related compounds, if they exist in nature, may be similarly difficult to identify. More recently the Lewinsohn group used a modified procedure to assess the capture of MR1-ligands from mammalian or insect cells infected with *E.coli* and *M. smegmatis* (29). LC-MS combined with molecular networking analysis revealed many candidate ligands, with a subset of these functionally validated using MR1T cell clones (29) (Table 1).

The chemical instability of MAIT antigens poses difficulties for the potential development of therapies targeting MAIT cells. On the other hand, this also provides us an insight into an important aspect of the biology of these cells. MAIT cells appear to be precisely poised to detect metabolically active microbes by recognizing antigens that are swiftly captured by MR1. Nevertheless, a few groups have attempted to develop tools in the form of more stable analogs, with the hope that these may better stimulate MAIT cell responses. Mak et al. reported that 5-OP-RU synthesized in DMSO had improved stability. Stabilized compounds (Figure 3) could be generated by replacing the exocyclic nitrogen atoms with carbon atoms (44, 46). However, whilst replicating a similar MAIT recognition and response, this analog was ~1,000-fold less efficient than 5-OP-RU in boosting MAIT cells numbers when delivered intranasally with CpG (46). This again, tells us something of the exquisite specificity of MR1-MAIT recognition, selected through the co-evolution of mammalian hosts and pathogenic or commensal microorganisms.

Using a different approach to tackle the chemical instability of MAIT antigens, the Painter group recently created a stabilized 5-A-RU “pro-drug” which releases 5-A-RU upon enzymatic cleavage in the recycling endosomes and which was active *in vivo* (123). Another group reported that the stability of the precursor 5-A-RU, long known as a chemically unstable precursor of riboflavin synthesis is increased by synthesizing and storing it as its HCl-salt (142). The recent availability of 5-A-RU from commercial sources should further facilitate its study in this context.

### Mouse Models

The ability of compounds to bind MR1, and to activate or inhibit MAIT cells, has been tested in various assays. However, understanding their physiological relevance requires *in vivo* models. Historically, the study of MAIT cells in mice has been difficult, due to their low numbers, and lack of specific reagents. Four main types of mice have been employed for their study; gnotobiotic (germ-free) mice, in which MAIT cells are barely detectable (12, 55), MR1^−/−^ mice, which do not develop MAIT cells (12), and conversely, transgenic mice expressing the Vα19*i* invariant TCR α chain (12, 143, 144), and CAST/EiJ mice (145), which have higher numbers of MAIT cells. The use of these and other tools to study MAIT cells in mice has been previously reviewed (83). With our recent understanding that MR1T cells exhibit greater diversity than originally believed, it is important to note that many studies use MR1^−/−^ mice as a “MAIT-cell deficient” model, but these will also lack MR1-dependent responses by non-MAIT MR1T cells. Additionally, the Vα19*i*Tg mice, are not a clear-cut “MAIT cell transgenic.” In these mice, MR1-tetramer-reactive cells were of high frequency, however about one third of these cells did not express the promyelocytic leukemia zinc finger (PLZF) protein, a hallmark of classical MAIT cells. Additionally, a high frequency of MR1-tetramer-reactive cells could be detected even in the absence of MR1 (Vα19*i*Tg.MR1^−/−^ mice) (54), and presumably these were selected by conventional MHC molecules during their development in the thymus. More recently, it has been described that MAIT cells are deficient in TCR Jα18 knockout mice (146), and also in Jα33 knockout mice, which revealed a residual population of MAIT-like TRAV1-2^+^ or TRAV1-2^−^ cells that were responsive to antigen or bacterial infection (59).

Models of infection with bacterial pathogens in mice (32–34, 54) and non-human primates (147) have revealed much about MAIT cell biology and their role in protective immunity. The development of MR1-5-OP-RU-tetramer reagents (7, 11) has enabled the specific assessment of MAIT cells in naïve SPF-housed mice (67, 130, 148), in disease-relevant models including infection, autoimmune disease (35, 149, 150), transplantation (151) and cancer (152), and MAIT cell development (55, 71, 153). MR1-tetramers also allow the assessment of the ability of defined compounds to act as antigens, with 5-OP-RU (67, 93), premixed 5-A-RU and methylglyoxal (25, 123) and a pro-drug designed to release 5-A-RU (123) all shown to boost MAIT cell numbers in mice, in the presence of TLR agonists or other co-stimuli. However, the direct assessment of MAIT cell responses to MR1 ligands *in vivo* is still a relatively under-explored area with more research needed.

## Conclusions

The MR1-MR1T cell field has progressed rapidly in the short time since the discovery of the first MR1-ligands. We now understand that both MR1-ligands and MR1-reactive T cells are more diverse than first believed. MR1T, particularly MAIT cells, have been studied in physiological settings such as homeostasis, infection, autoimmune disease and cancer, sometimes with contradictory findings in both human cohorts and animal studies. There remain several questions and challenges in the field. For example, we do not fully understand the production of ligands from microbes, how these enter cells, how they are processed and presented, or the full range of signals determining the quality and quantity of the TCR-MR1-dependent response. Deciphering the complexity of antigen presentation and recognition, and the relative importance of other signals contributing to the MR1T response, particularly within the local tissue environment, is essential to understanding their biology. For instance, how do these cells differentiate between pathogens and commensal microorganisms allowing them to play diverse roles including aiding the clearance of pathogens, tissue repair and gut homeostasis? It is possible that there is a threshold amount of antigen that needs to penetrate the mucosal barriers before they will respond, and thus the abundant microbes in the intestines, which can synthesize riboflavin, are normally ignored. However, we consider it likely that the context of antigen and other signals drives different responses, including in a tissue-specific manner, whereby these cells contribute to homeostasis or inflammation and immune protection. Answering these questions will require the analysis of clearly defined MR1T subsets, and likely new tools to combat the issues of chemical instability and ligand identification. Additionally, the definition of an increasing number of MR1T subsets with different Ag-recognition and phenotypic features raises many questions about the physiological relevance and therapeutic potential of these cells. MR1T cells offer great opportunities as attractive targets in vaccination (154) and immunotherapy (61). We hope that further advances in understanding T cell recognition of MR1-ligands using definitive tools and approaches such as those described above, will enable the MR1-MR1T axis to be harnessed to combat infection and disease.

## Author Contributions

WA and AC generated the figures and tables. All authors wrote and edited the manuscript.

## Conflict of Interest

AC and ZC are inventors on patents describing MR1 ligands and MR1-tetramer reagents. The remaining authors declare that the research was conducted in the absence of any commercial or financial relationships that could be construed as a potential conflict of interest.
